# The lysin motif-containing proteins, Lyp1, Lyk7 and LysMe3, play important roles in chitin perception and defense against *Verticillium dahliae* in cotton

**DOI:** 10.1186/s12870-017-1096-1

**Published:** 2017-09-04

**Authors:** Jun Xu, Guilin Wang, Jing Wang, Yongqing Li, Liangliang Tian, Xinyu Wang, Wangzhen Guo

**Affiliations:** 1 State Key Laboratory of Crop Genetics & Germplasm Enhancement, Nanjing Agricultural University, Nanjing, 210095 Jiangsu Province People’s Republic of China; 2 College of Life Sciences, Nanjing Agricultural University, Nanjing, 210095 Jiangsu Province People’s Republic of China

**Keywords:** Lysin motif, Pattern recognition receptors, Plasma membrane localization, Chitin signals, *Verticillium dahliae*, Cotton

## Abstract

**Background:**

Lysin motif (LysM)-containing proteins are important pattern recognition receptors (PRRs) in plants, which function in the perception of microbe-associated molecular patterns (MAMPs) and in the defense against pathogenic attack. To date, the LysM genes have not been systematically analyzed in cotton or effectively utilized for disease resistance.

**Results:**

Here, we identified 29, 30, 60, and 56 LysM genes in the four sequenced cotton species, diploid *Gossypium raimondii*, diploid *G. arboreum*, tetraploid *G. hirsutum* acc. TM-1, and *G. barbadense* acc. 3–79, respectively. These LysM genes were classified into four groups with different structural characteristics and a variety of expression patterns in different organs and tissues when induced by chitin or *Verticillium dahliae*. We further characterized three genes, *Lyp1*, *Lyk7* and *LysMe3*, which showed significant increase in expression in response to chitin signals, *V. dahliae* challenge and several stress-related signaling compounds. Lyp1, Lyk7 and LysMe3 proteins were localized to the plasma membrane, and silencing of their expression in cotton drastically impaired salicylic acid, jasmonic acid, and reactive oxygen species generation, impaired defense gene activation, and compromised resistance to *V. dahliae*.

**Conclusion:**

Our results indicate that Lyp1, Lyk7, and LysMe3 are important PRRs that function in the recognition of chitin signals to activate the downstream defense processes and induce cotton defense mechanisms against *V. dahliae*.

**Electronic supplementary material:**

The online version of this article (10.1186/s12870-017-1096-1) contains supplementary material, which is available to authorized users.

## Background

Various immune responses are triggered in plants against invading pathogens upon the perception of so-called pathogen associated molecular patterns (PAMPs) at the plant cell surface. Most of the known PAMPs have been well-characterized as either polypeptide type or carbohydrate type, such as two ligands, Flg22 (a peptide representing the elicitor-active epitope of the bacterial flagellin) and Elf18 (an EF-Tu-derived peptide). And their interrelated receptor proteins FLS2 and EFR have been identified as the leucine-rich repeat receptor-like kinases (LRR-RLKs) in *Arabidopsis thaliana* [1, 2]. Chitin, a major component of the fungal cell wall, is a well-known PAMP that can be recognized by pattern recognition receptors (PRRs) at the plant cell surface and activate PAMP-triggered immunity (PTI) [3]. PTI includes the activation of pathogenesis-related (PR) genes and the production of reactive oxygen species (ROS) [4]. In addition, chitin binding sites and plasma membrane receptor proteins have been detected in membrane preparations of different plants, and have been shown to activate downstream defense processes [5–8].

The Lysin motif (LysM) domain, a protein module that recognizes chitooligosaccharides, peptidoglycan and other related N-acetylglucosamine (GlcNAc)-containing oligosaccharides, which usually contains about 40 amino acids (AA) and is a ubiquitous modular cassette that exists in all living organisms except for Archaea [9, 10]. In plants, a chitin elicitor binding protein, CEBiP, was first reported to be a LysM domain-containing protein and involved in the binding and perception of chitooligosaccharides in rice [3]. Subsequently, LysM-encoding genes were identified in a broad range of organisms at a genome-wide level using the increasing numbers of transcript and genomic sequences. According to the subcellular location and domain structure of LysM genes, members of the family can be divided into four subgroups; LysM-containing receptor-like kinases (Lyks), LysM-type receptor-like proteins (Lyps), extracellular LysM proteins (LysMes) and nonsecretory intracellular LysM proteins (LysMns) [10, 11]. The multiple domains and the complex structures of the LysM genes are indicative of the variety of functions they carry out.

As PAMP receptors, LysM domain-containing proteins can sense bacterial oligosaccharides, peptidoglycan (PGN) and fungal chitin, and respond by promoting the plant’s defenses. In *Arabidopsis*, Lyks are defined as receptors for chitin, and hence, are implicated in plant defense mechanisms against fungal pathogens [12]. AtCERK1, which is a cell surface chitin elicitor receptor kinase 1, directly binds chitin through its LysM-containing ectodomain (AtCERK1-ECD) to trigger immune responses [13, 14]. At LYK4 also mainly assists chitin signal transduction and activates plant innate immunity through chitin recognition [15]. In addition, AtLYK5 not only shares functions with AtLYK4 in mediating the chitin response, but also binds to chitin with a higher affinity than AtCERK1. AtLYK5 is also necessary for chitin-induced AtCERK1 phosphorylation and homodimerization. AtCERK1, AtLYK4 and AtLYK5 are membrane-localized proteins, and are all involved in plant responses to chitin [16]. Two rice LysM receptor molecules, CEBiP and CERK1, have been identified as critical in the regulation of chitin elicitor signaling [17]. Moreover, two LysM receptor proteins, LYP4 and LYP6, have also been identified as peptidoglycan and chitin perception receptors in rice [18]. To date, however, the functions of the LysM genes in cotton remain largely unknown.

*Verticillium dahliae*, a destructive soil-borne fungal pathogen, causes cotton *Verticillium* wilt and leads to severe reductions in cotton yield across the world [19]. Although huge efforts have been made to generate wilt-resistant cotton cultivars through traditional breeding, this remains a challenge [20]. Recently, progress has been made in excavating PR genes and exploring the molecular mechanisms of responses to *V. dahliae* invasion in cotton. Several *V. dahliae*-responsive genes, such as *GhNDR1*, *GhNaD1*, *GbWRKY1*, *GhSSN*, and *GhMLP28,* have been shown to be functionally related to defense responses to *V. dahliae* attack in cotton [21–25]. Nevertheless, there is an urgent requirement to mine more candidate genes in order to develop *Verticillium* wilt-resistant cotton cultivars.

The availability of data on the whole-genome of different *Gossypium* cotton species, including *G. raimondii* (D5) [26], *G. arboreum* (A2) [27], *G. hirsutum* acc. TM-1 (AD1) [28], and *G. barbadense* acc. 3–79 (AD2) [29], has made it possible to systematically identify and analyze the targeted genes on a genome-wide level, and has thus enriched our understanding on the molecular mechanisms of cotton responses to *V. dahliae* invasion. In the present study, we systematically surveyed LysM members in four sequenced cotton species for the first time, and analyzed their phylogenetic relationships, gene structures, subcellular localizations and expression patterns in different tissues in control conditions, and in response to PAMPs, *V. dahliae* and different stress-related signaling compounds. Furthermore, we found that silencing of *Lyp1*, *Lyk7*, and *LysMe3* expression in cotton significantly impaired tolerance of *V. dahliae*, indicating that these genes played important roles in the defense response mediated by chitin recognition. Moreover, down-regulation of *Lyp1*, *Lyk7*, and *LysMe3* expression hampered downstream resistance-related pathways and the activation of PR genes. The study not only enriches our knowledge of the networks involving in LysMs in cotton, but also provides effective gene resources for the development of *Verticillium* wilt-resistant cultivars through cotton-breeding programs.

## Results

### Genome-wide identification and characterization of LysM genes

We used HMMER 3.0 and the domain “lysin” from the Pfam protein family database (PF01476) to search for LysM genes in four released genome sequences; diploid *G. raimondii* and *G. arboreum*, and tetraploid *G. hirsutum* and *G. barbadense*. Subsequently, we used the SMART [30] and INTERPROSCAN [31] programs to verify the predicted genes. As a result, 29, 30, 60, and 56 LysM genes were identified in *G. raimondii*, *G. arboreum*, *G. hirsutum* acc. TM-1, and *G. barbadense* acc. 3–79, respectively (Additional file 1: Table S1).

**Fig. 1:**
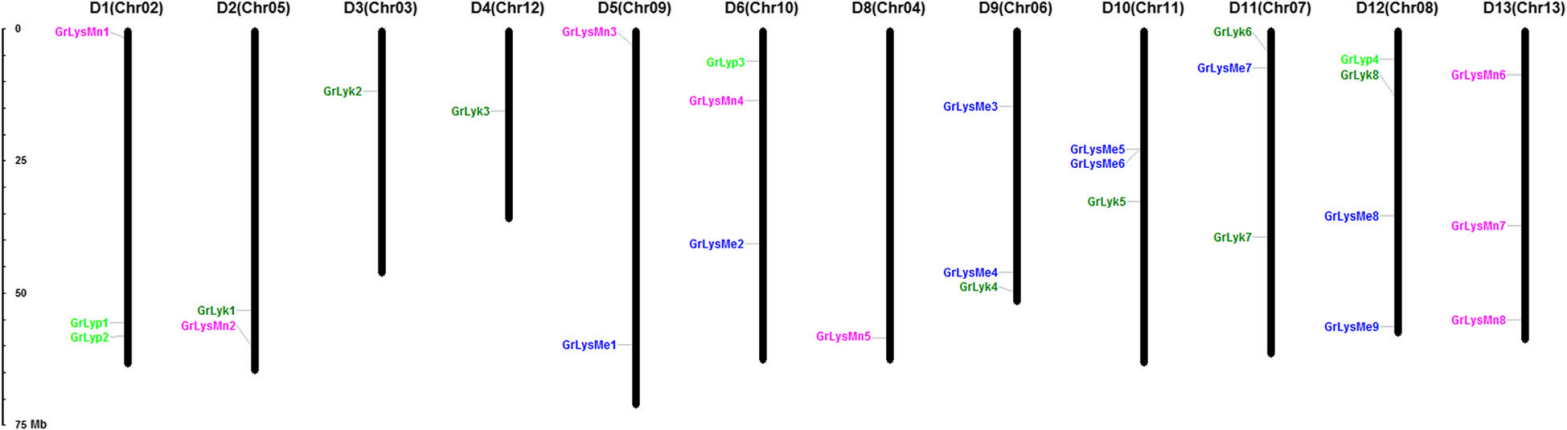
Chromosomal distribution of LysM genes in *G. raimondii.* The chromosome numbers are shown at the top of each bar. The 29 LysM genes in *G. raimondii* were classified into four groups and marked on the linkage map. The names of the scaffolds from the genome are also indicated in brackets. The chromosome numbers from D1 to D6, and D8 to D13, were consistent with the newly-updated interspecific genetic map of allotetraploid cultivated cotton species [32]. The nomenclature of the LysM genes for each group was based on the order of the chromosomes in *G. raimondii*

Based on the conserved domains, motif components and exon-intron organization of the LysM genes, we classified them into four categories; *Lyk*s, *Lyp*s, *LysMe*s and *LysMn*s (Additional file 2: Figure S1). The LysM genes were relatively conserved within each group, but obvious differences existed between groups. To elucidate the chromosomal distribution of these LysM genes, we integrated 13 scaffolds of the *G. raimondii* genome (named Chr01 to Chr13) [26] into the previously reported high-density interspecific genetic map of allotetraploid cultivated cotton species [32], and then reordered the 13 *G. raimondii* scaffolds according to the corresponding D1 to D13 chromosomes of the tetraploid cotton species (Fig. 1). Following the new order of the *G. raimondii* chromosomes, the LysM genes in *G. raimondii* were named *GrLyp1* to *GrLyp4*, *GrLyk1* to *GrLyk8*, *GrLysMe1* to *GrLysMe9*, and *GrLysMn1* to *GrLysMn8* (Fig. 1). The 29 LysM genes in *G. raimondii* were matched to 12 scaffolds, and no genes were matched to the D7 chromosome scaffold. The chromosomal distribution of the LysM members was uneven, with chromosomes D1, D6, D9, D10, D11, D12 and D13 containing more than three genes each, and the other chromosomes containing smaller numbers of LysM genes. Furthermore, the corresponding orthologs in three other sequenced cotton species, *G. arboreum*, *G. hirsutum* acc. TM-1, and *G. barbadense* acc. 3–79, were named respective to the orthologs in *G. raimondii* (Additional file 1: Table S1).

Two publicly available algorithms, SignalP and TargetP, were used to predict the subcellular locations of these LysM proteins. SignalP predicted that 15 of the 29 LysM proteins contained signal peptides, meanwhile TargetP analyses predicted that 18 of these proteins were involved in secretory pathways. In addition, SMART programs confirmed that 12 of the LysM proteins contained transmembrane domains (Additional file 3: Table S2). These findings indicate that LysM proteins are involved in MAMP recognition and therefore perform crucial roles in plant defense responses.

### Expression analysis of LysM genes in response to two PAMPs and *V. dahliae*

**Fig. 2:**
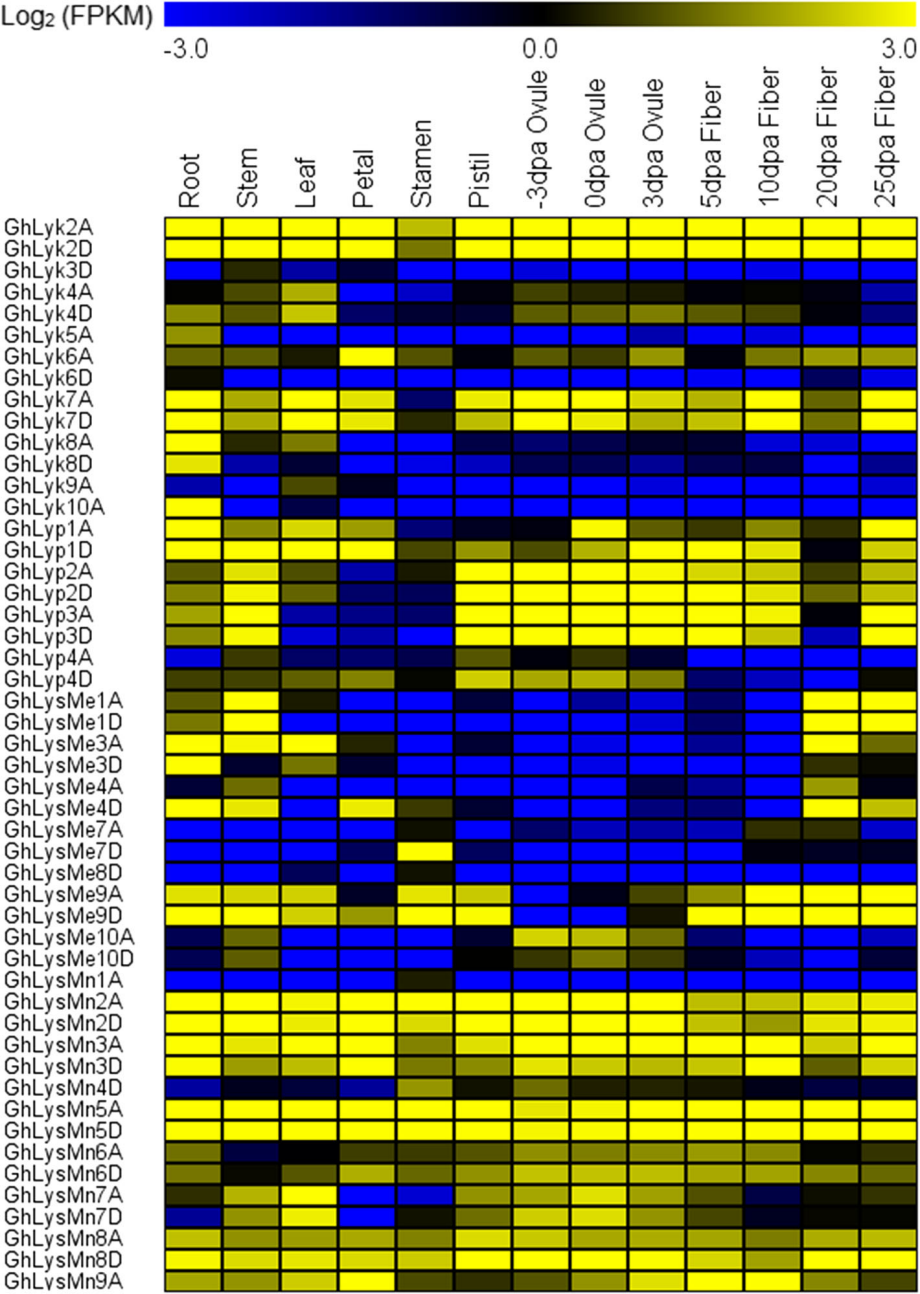
Transcriptional profiling of LysM genes in different tissues and organs of *G. hirsutum* acc. TM-1. Roots, stems, leaves, petals, stamens, ovules at −3, 0, and 3 DPA, and fibers at 5, 10, 20, and 25 DPA were used for comparative transcriptome analysis. The expression data were converted to Log_2_ (FPKM) to calculate the expression levels of the LysM genes in TM-1. Differences in gene expression are shown in the colors indicated in the scale. The RNA-Seq data used here can be downloaded from http://www.ncbi.nlm.nih.gov/bioproject/PRJNA248163/

**Fig. 3:**
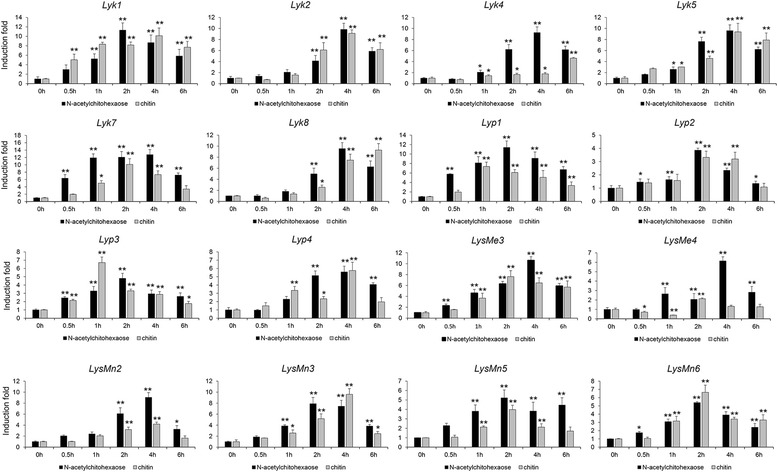
Induced expression of LysM genes by two PAMPs. Two-week-old seedlings were treated with either 200 mg/mL insoluble crab shell chitin or soluble chitin fragment N-acetylchitohexaose and sampled 0.5, 1, 2, 4, 6 h (h) after treatment, respectively. The induction of each gene was examined by qRT-PCR, and the 0 h expression levels were used as controls when calculating the level of induction. The data represent the mean ± SD of three samples from three independent tests at each time point. “*”: significant difference at *P* < 0.05; “**”: significant difference at *P* < 0.01

To further investigate the roles of LysM genes in tetraploid cotton, we used transcriptome data from *G. hirsutum* acc. TM-1 vegetative tissues (root, stem, and leaf), floral tissues (petal and anther), and ovule and fiber tissues at −3, 0, 3, 5, 10, 20, and 25 days post anthesis (DPA) to determine the expression patterns of these genes [28]. A total of 50 LysM genes were expressed in *G. hirsutum* acc. TM-1 with FPKM > 1.0, and the developmental and spatial regulation of these genes differed between the various tissues (Fig. 2). As previously reported, many LysM genes played important roles in chitin recognition, chitin signal transduction and the activation of plant innate immunity [13–15, 18, 33]. To better understand the function of LysM genes, we selected 20 genes with relatively high expression levels in the root tissue and analyzed their expression when induced by *V. dahliae* and by two PAMPs, insoluble crab shell chitin and soluble chitin fragment N-acetylchitohexaose. In detail, the transcripts of 16 LysM genes, 6 *Lyk*s, 4 *Lyp*s, 2 *LysMe*s, and 4 *LysMn*s, were significantly upregulated following treatment with two PAMPs (Fig. 3). These 16 LysM genes exhibited maximal expression levels 1 h, 2 h or 4 h after treatment, and both insoluble crab shell chitin and soluble chitin fragment N-acetylchitohexaose treatment caused a 6- to 13-fold increase in their expression. Significantly, *Lyk7* of the Lyk category had a higher expression level than other members; reaching maximal levels of a 13-fold increase 4 h after N-acetylchitohexaose treatment, and a 10-fold increase 2 h after chitin treatment. The expression of *Lyp1* in the *Lyp*s category was significantly upregulated following treatment, with increases of 11- and 7-fold 2 h after N-acetylchitohexaose treatment and 1 h after chitin treatment, respectively. In the *LysMes* category, an 11-fold increase in *LysMe3* transcript levels was induced 4 h after N-acetylchitohexaose treatment, and an 8-fold increase was seen 2 h after chitin treatment. In the *LysMn*s category, the expression of the *LysMn2* was increased by 9- fold at 4 h after N-acetylchitohexaose treatment, but the peak values of *LysMn3*, *LysMn5*, and *LysMn6*, of 7.8-, 5.2-, and 5.4- fold increases, respectively, were observed 2 h after treatment. In addition, the expression levels of *LysMn2* and *LysMn3* increased 4.2- and 9.6-fold 4 h after chitin treatment; however, the level of *LysMn5* and *LysMn6* transcript expression increased more quickly, with respective peaks of 3.9- and 6.6-fold increases reached 2 h after treatment. These results suggest a role for LysM genes in chitin recognition; an important part of cotton innate immunity.

**Fig. 4:**
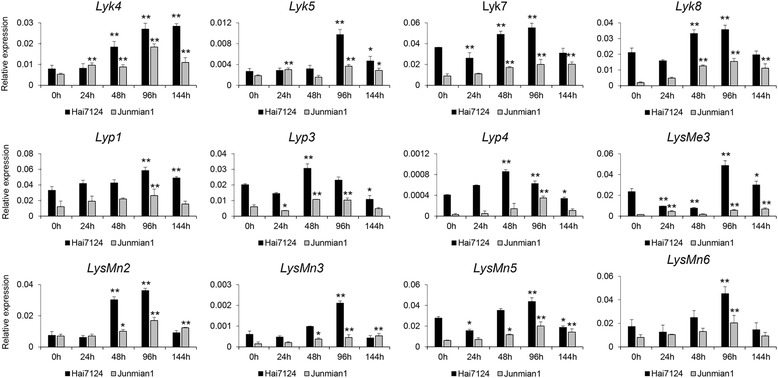
Expression patterns of the LysM genes in response to *Verticillium dahliae* in cotton. The expression patterns of LysM genes in response to *Verticillium dahlia* were investigated in *G. barbadense* cv. Hai7124 and *G. hirsutum* cv. Junmian 1, which show resistance and susceptibility to *V. dahliae*, respectively. qRT-PCR analysis showed differences in the expression of LysM genes in Hai7124 and Junmian 1 after inoculation with *V. dahliae* strain V991. The statistical analysis compared expression levels at different time points following treatment with those at 0 h. Error bars show the standard deviation of three biological replicates. “*”: significant difference at *P* < 0.05; “**”: significant difference at *P* < 0.01

Plant receptor kinases and receptor proteins containing lysin motifs play crucial roles in the recognition of carbohydrate patterns commonly associated with microbial surfaces and in the defense against microbial infection [3, 12, 14–17, 33]. To better ascertain the functions of LysM proteins in plant defenses against *V. dahliae*, we analyzed the expression patterns of 16 LysM genes after *V. dahliae* inoculation in Hai7124 and Junmian 1, which exhibited resistance and susceptibility to *V. dahliae*, respectively. Overall, the expression levels of these LysM genes were higher in Hai7124 than in Junmian 1. Further, 12 LysM genes, 4 *Lyk*s, 3 *Lyp*s, 1 *LysMe*, and 4 *LysMn*s, were significantly induced by *V. dahliae* and quickly reached peak expression levels at different time points in Hai7124 (Fig. 4). Among them, *Lyk*7, *Lyp1*, *LysMe*3, and *LysMn*6 showed the highest expression levels of their respective groups after *V. dahliae* treatment, suggesting that these genes played important roles in chitin recognition and defense against *V. dahliae*.

### Silencing *Lyp1*, *Lyk7*, and *LysMe3* impairs cotton tolerance to *V. dahliae*

To obtain further evidence of the role of LysM genes in *V. dahliae* resistance, we selected *Lyp1*, *Lyk7*, *LysMe3*, and *LysMn6,* which had the highest expression levels of their groups and were significantly induced after chitin, chitin fragment N-acetylchitohexaose, and *V. dahliae* treatments, for functional identification via virus-induced gene silencing (VIGS) analysis.

We constructed TRV: *GbLyp1*, TRV: *GbLyk7*, TRV: *GbLysMe3* and TRV: *GbLysMn6* vectors to silence endogenous genes in Hai7124, and used TRV: 00 as a mock treatment and TRV: *GhCLA1* as a positive control to validate the efficiency of the VIGS assay. As expected, the cotton leaves showed an obvious photobleaching phenotype 2 weeks after agroinfiltration with the *GhCLA1* construct (Additional file 4: Figure S2), indicating that the VIGS system worked well in our experimental operations. The cotton seedlings that were confirmed to be infiltrated with one of the constructs and those with the mock treatment were selected for RNA isolation and quantitative real-time PCR (qRT-PCR) analysis. Hai7124 and Junmian 1 plants were used as controls resistant to and susceptible to *V. dahliae*, respectively, and were challenged with *V. dahliae* strain V991 by the dip-infection method at a final concentration of 1 × 10^7^ spores per milliliter (ml) [34]. About 10 days after inoculation, the seedlings of the Junmian 1 plants showed obvious cotyledon wilting, but only a small number of Hai7124 plants displayed the leaf wilting phenotype and these only appeared at least 15 days after inoculation with *V. dahliae*. These results further confirmed that Hai7124 and Junmian 1 plants were resistant and susceptible to *V. dahliae*, respectively, and could be used as test cultivars to investigate the virulence of *V. dahliae* in cotton. The phenotypes of the two control plants at 20 and 25 days after inoculation with *V. dahliae* are shown in Additional file 5: Figure S3.

**Fig. 5:**
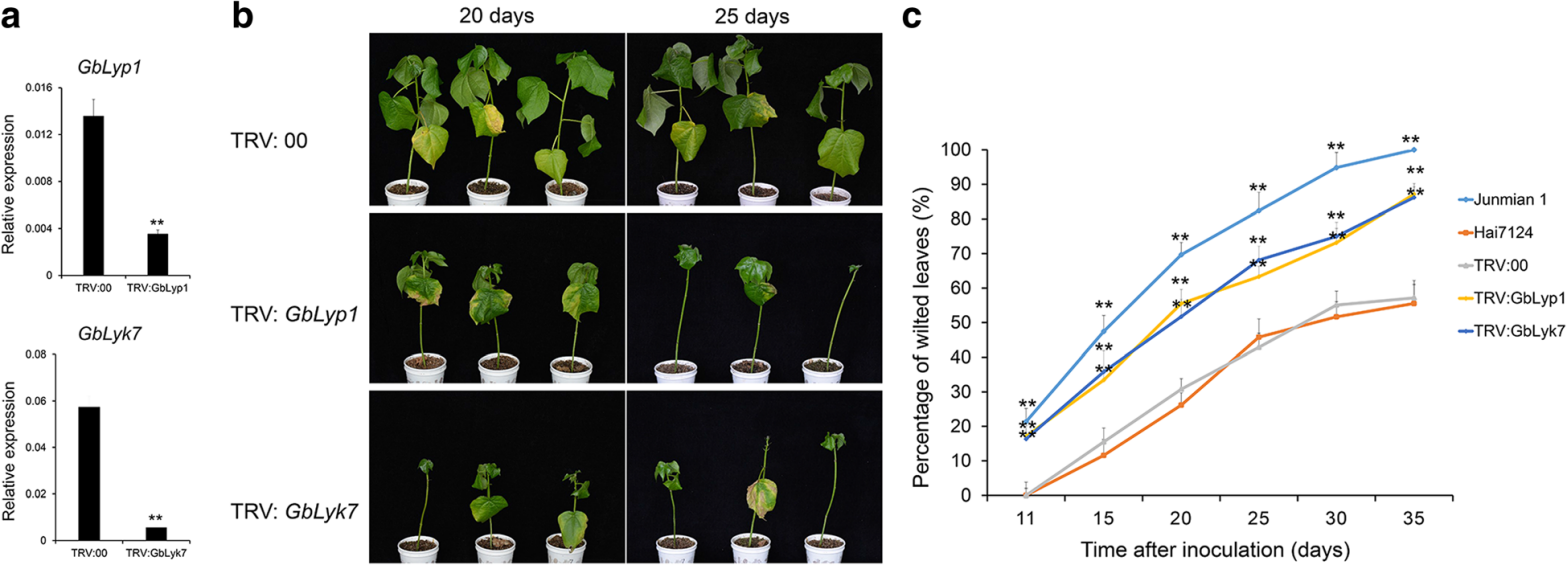
Silencing of *Lyp1* and *Lyk7* significantly impaired the resistance to *V. dahliae* in *G. barbadense* cv. Hai7124. *Lyp1* and *Lyk7* were silenced in *V. dahliae* resistant Hai 7124 seedlings by VIGS, and about 2 weeks later, the seedlings were inoculated with *V. dahliae* at a concentration of 1 × 10^7^ spores/mL. **a** Analysis of *Lyp1* and *Lyk7* expression levels. Total RNA was extracted from the leaves of the VIGS seedlings 14 d post-agroinfiltration, and transcription levels of *Lyp1* and *Lyk7* in the *Lyp1* and *Lyk7*-silenced plants were compared with that of the control (TRV: 00 plants). Asterisks indicate statistically significant differences, as determined by Student’s t-tests (**P < 0.01). **b** Disease symptoms of the *Lyp1* and *Lyk7*-silenced plants 20 and 25 days after *V. dahliae* inoculation; **c** The percentage of diseased leaves of the *Lyp1* and *Lyk7*-silenced plants and controls after *V. dahliae* inoculation. These experiments were repeated using at least 20 seedlings per treatment. Error bars show the standard deviation of three biological replicates. Asterisks indicate statistically significant differences in the percentage of diseased leaves between treated plants and TRV: 00 controls, as determined by Student’s t-tests (*P < 0.05, **P < 0.01)

Compared with the controls, the expression levels of *Lyp*1 and *Lyk*7 were significantly lower in *Lyk7*- and *Lyp1*-silenced plants (Fig. 5a); however, the transcript levels of the other genes in the same group were not changed in the VIGS plants (Additional file 6: Figure S4). Further, the *Lyp1*- and *Lyk7*- silenced plants were inoculated with *V. dahliae*. No significant phenotypic differences were observed between the VIGS plants and Hai7124 control plants before pathogen invasion. However, 15 days after infection, the VIGS plants began to show obvious leaf-yellowing phenotypes, and 20 or 25 days after *V. dahliae* invasion, the *Lyp1*-, and *Lyk7*-silenced plants displayed more severe wilting and yellowing symptoms and more etiolated leaves than the control plants, suggesting that the *Lyp1*-, and *Lyk7*-silenced plants had a higher susceptibility to *V. dahliae* (Fig. 5b). After a further 10 days, almost all the true leaves were defoliated in *Lyp1*- and *Lyk7*-silenced plants, meanwhile, a similar phenotype was observed in Junmian 1 at 25 days after *V. dahliae* invasion (Additional file 5: Figure S3). To better investigate the susceptibility of *Lyp1*- and *Lyk7*-silenced plants to *V. dahliae*, more than 20 plants per treatment were used to calculate the ratio of diseased to non-diseased leaves. The Hai7124 seedlings without injection and TRV: 00 control plants had similar characteristics, including few wilted leaves and a ratio of diseased leaves to healthy leaves of approximately 50% 35 days after inoculation. However, about 85% of the *Lyp*1- and *Lyk*7-silenced plants displayed leaf wilting or defoliation, and nearly 100% disease was observed in the susceptible control Junmian 1 plants 35 days after inoculation (Fig. 5c, Additional file 7: Table S3).

**Fig. 6:**
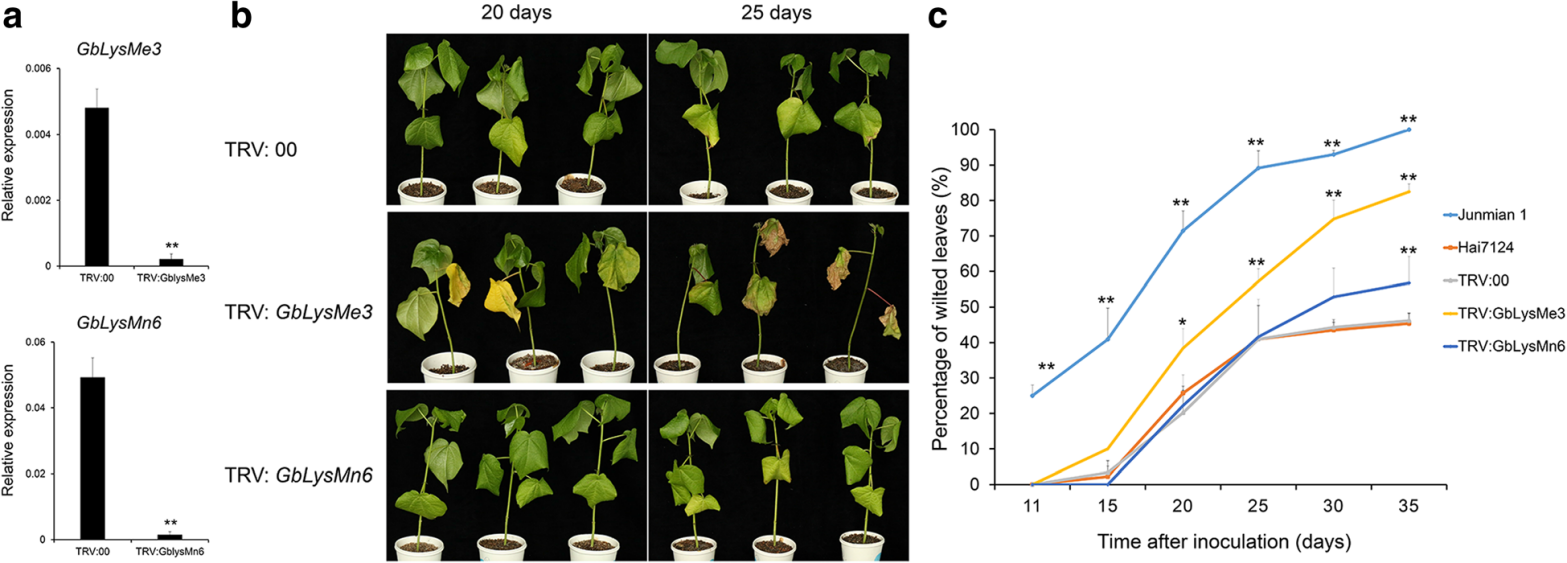
Increased susceptibility of the *LysMe3*- and *LysMn6*- silenced cotton plants to *V. dahliae*. *LysMe3* and *LysMn6* were silenced by VIGS in Hai7124 seedlings, and about 2 weeks later, the seedlings were inoculated with *V. dahliae* at a concentration of 1 × 10^7^ spores/mL. **a** The expression levels of *LysMe3* and *LysMn6* were compared in the *LysMe3*-*, LysMn6*- silenced and TRV: 00 cotton plants*.* Asterisks indicate statistically significant differences, as determined by Student’s t-tests (**P < 0.01). **b** Phenotypes of the *LysMe3*-*, LysMn6*-silenced plants 20 and 25 days after *V. dahliae* inoculation. **c** The percentage of diseased leaves in the *LysMe3*-, and *LysMn6*-silenced plants and controls after *V. dahliae* inoculation. All experiments were repeated using at least 20 seedlings, and error bars show the standard deviation of three biological replicates. Asterisks indicate statistically significant differences in the percentage of diseased leaves between treated plants and TRV: 00 controls, as determined by Student’s t-test (*P < 0.05, **P < 0.01)

In parallel, we also performed qRT-PCR to analyze the expression of *LysMe*s and *LysMn*s in the *LysMe3*- and *LysMn6*- silenced plants, respectively. Only *LysMe3* and *LysMn6* were significantly down-regulated in the VIGS plants compared to the TRV: 00 control plants (Fig. 6a, Additional file 6: Figure S4). The *LysMe3*- and *LysMn6*- silenced plants were inoculated with *V. dahliae*. Down-regulation of *LysMe3* resulted in impaired tolerance to *V. dahliae*, yet there were no significant changes in tolerance to *V. dahliae* in the *LysMn6*-silenced plants compared to controls (Fig. 6b). The percentage of diseased leaves in *LysMe3*-silenced plants was consistently higher than in controls and reached 80% 35 days after inoculation, but in LysMn6-silenced plants, only 56% of leaves displayed wilting or defoliation, which was similar to that in TRV: 00 and Hai 7124 plants (Fig. 6c, Additional file 8: Table S4).

Taken together, these results show that silencing *Lyp1*, *Lyk7*, and *LysMe3* in cotton plants significantly increases their susceptibility to *V. dahliae*, suggesting that these three genes play important roles in the resistance to *V. dahliae* infection.

### Lyp1, Lyk7 and LysMe3 are located in the plasma membrane

**Fig. 7:**
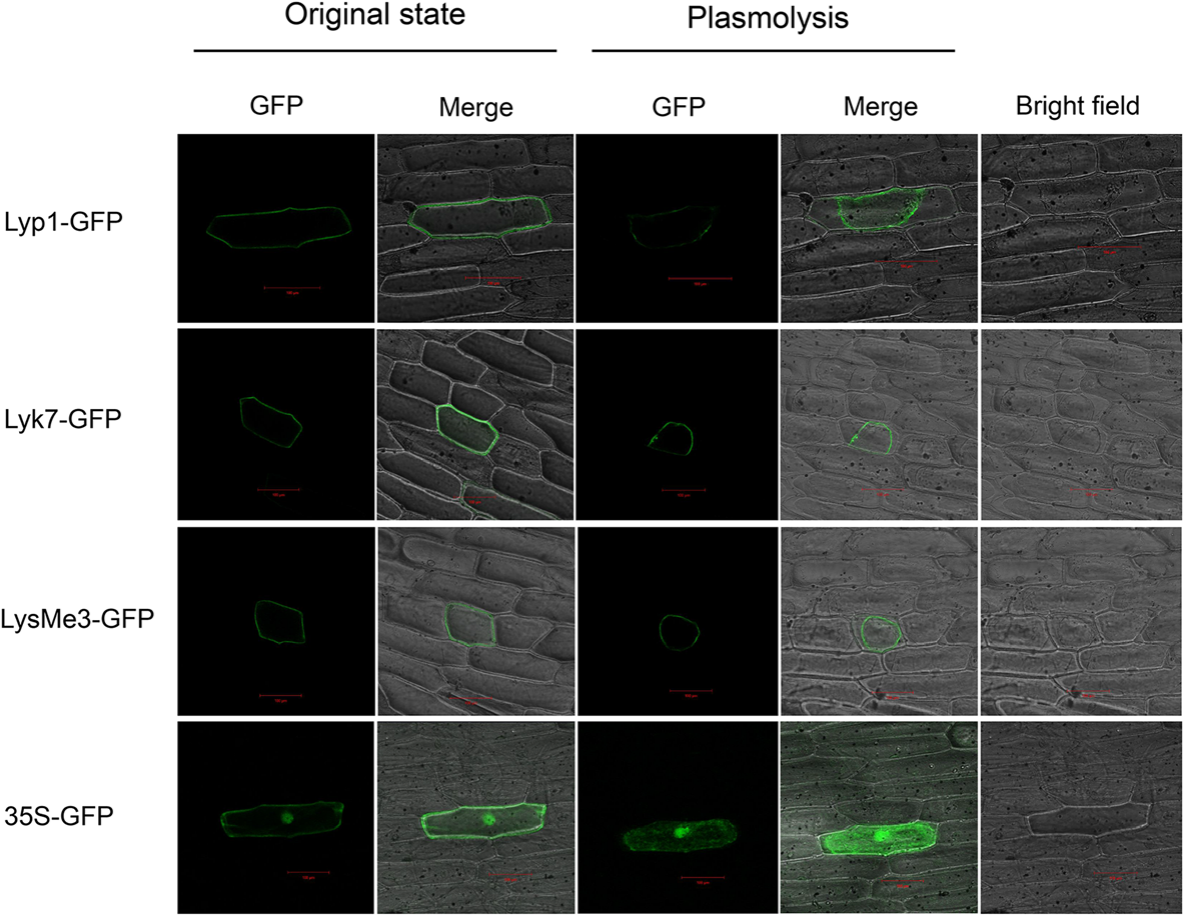
Subcellular localization of Lyp1, Lyk7, and LysMe3 proteins. The GFP, *Lyp1*-GFP, *Lyk7*-GFP, and *LysMe3*-GFP fusion proteins were transiently expressed in onion epidermal cells. GFP fluorescence was visualized by confocal microscopy, and 20% sucrose solution was used for the plasmolysis of the onion cells. Scale bars = 100 μm

To gain direct evidence of the subcellular localization of Lyp1, Lyk7 and LysMe3, we transiently expressed these proteins fused to GFP in onion epidermal cells using biolistic bombardment. A pBIN-GFP4 vector was used as a control and fluorescence was detected under a confocal microscope. As shown in Fig. 7, fluorescence from the Lyp1, Lyk7 and LysMe3 GFP fusion proteins was detected in the cell membrane, and the shape of the membrane was changed during the plasmolytic processes. However, GFP control fluorescence (35S–GFP) was detected throughout the cell. Taken together, these findings suggest that Lyp1, Lyk7, and LysMe3 are located in the cell membrane, and these typically membrane-anchored proteins might be involved in recognizing PAMPs and activating a variety immune responses.

### *Lyp1*, *Lyk7*, and *LysMe3* are crucial for downstream defense reactions

**Fig. 8:**
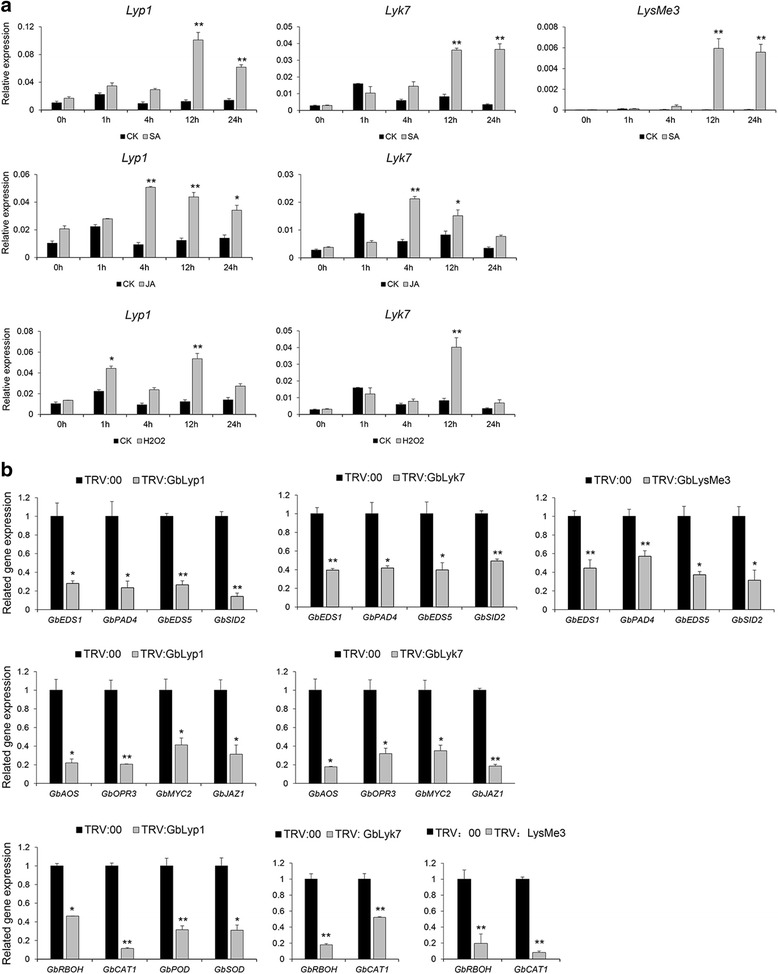
*Lyp1*, *Lyk7*, and *LysMe3* were involved in SA, JA, and ROS production*.* **a** Comparison of the expression levels of *Lyp1*, *Lyk7* and *LysMe3* in cotton plants treated with SA, JA, and ROS signal compounds and H_2_O-treated control plants. **b** The transcription levels of genes related to SA, JA, and ROS signaling pathways were analyzed in *Lyp1*, *Lyk7*, and *LysMe3*-silenced plants and TRV: 00 control plants by qRT-PCR. Error bars represent the standard deviation of three independent experiments with three technical replicates for each experiment. Asterisks indicate statistically significant differences, as determined by Student’s t-tests (*P < 0.05, **P < 0.01)

To better investigate the roles of *Lyp1*, *Lyk7*, and *LysMe3* in the cotton-*V. dahliae* interaction, we further analyzed their expression patterns in cotton treated with the defense-related signaling molecules, salicylic acid (SA), jasmonic acid (JA), ethylene (ET) and hydrogen peroxide (H_2_O_2_). As shown in Fig. 8a, *Lyp1*, *Lyk7*, and *LysMe3* were significantly upregulated following SA, JA, or H_2_O_2_ treatment at different time points. In detail, *Lyp1* and *Lyk7* were clearly induced by SA, JA, and H_2_O_2_ treatment, whereas *LysMe3* was only upregulated by treatment with SA. These findings hint at a potential involvement of *Lyp1*, *Lyk7*, and *LysMe3* in the SA, JA, and H_2_O_2_ signaling pathways.

Further, we analyzed the expression levels of several key genes involved in the SA (*GbEDS1*, *GbPAD4*, *GbEDS5*, and *GbSID2*) [35, 36], JA (*GbAOS*, *GbOPR3*, *GbMYC2*, and *GbJAZ1*) [37], and H_2_O_2_ (*GbRBOH*, *GbCAT1*, *GbPOD*, and *GbSOD*) [38, 39] pathways in *Lyp1-*, *Lyk7-*, and *LysMe3-* silenced cotton plants. Compared with TRV: 00 control plants, *GbEDS1*, *GbPAD4*, *GbEDS5*, and *GbSID2* transcripts were significantly downregulated in the *Lyp1*-, *Lyk7*-, and *LysMe3*- silenced cotton plants, meanwhile the expression of *GbAOS*, *GbOPR3*, *GbMYC2,* and *GbJAZ1* was also reduced in the *Lyp1*-, *Lyk7*- silenced plants. In addition, down-regulation of *Lyp1*, *Lyk7*, and *LysMe3* resulted in reduced expression of *GbRBOH* and *GbCAT1*, and *GbPOD* and *GbSOD* were also down-regulated in the *Lyp1*- silenced plants (Fig. 8b). Based on these results, *Lyp1*, *Lyk7*, and *LysMe3* might participate in the SA pathway and ROS production, and *Lyp1* and *Lyk7* may also be involved in JA generation and accumulation. These findings indicate that the three membrane-anchored proteins, Lyp1, Lyk7, and LysMe3, are responsible for activating downstream SA, JA, and ROS pathways as part of the cotton defense against *V. dahliae.*

**Fig. 9:**
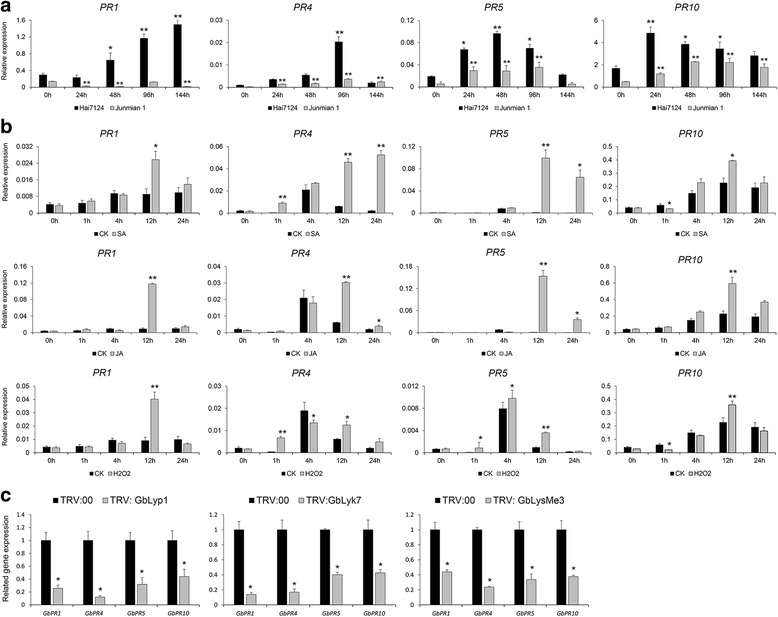
*Lyp1*, *Lyk7*, and *LysMe3* activated downstream defense genes. **a** The expression of defense-related genes (*PR1*, *PR4*, *PR5*, and *PR10*) was analyzed in Hai7124 and Junmian 1 after inoculation with *V. dahliae* strain V991. The 0 h treatments of the Hai7124 and Junmian 1 acted as controls. Asterisks indicate statistically significant differences, as determined by Student’s t-tests (*P < 0.05, **P < 0.01). **b** The transcript levels of the four PR genes in cotton treated with SA, JA, and H_2_O_2_ were detected, with H_2_O treatment acting as a control. **c** The expression levels of the defense genes were compared in the TRV: 00 control and VIGS cotton plants. Error bars represent the standard deviation of three independent experiments with three technical replicates for each experiment. Asterisks indicate statistically significant differences, as determined by Student’s t-tests (*P < 0.05, **P < 0.01)

As previously reported [40–43], the PR genes play important roles in signal recognition and plant immunity. To identify the PR proteins involved in cotton defense mechanisms against *V. dahliae*, we analyzed the expression patterns of *PR1* [40], *PR4* [41], *PR5* [42], and *PR10* [43] in Hai7124 and Junmian 1 after treatment with *V. dahliae* strain V991. As shown in Fig. 9a, these four genes were significantly induced in the two cotton cultivars after *V. dahliae* infection, however, the expression levels were higher in Hai7124 than in Junmian 1, suggesting that these genes play important roles in protecting cotton plants against *V. dahliae* infection. In addition, the levels of transcripts of these four defense-related genes were upregulated in cotton following exogenous application of SA, JA, and ROS (Fig. 9b).

**Fig. 10:**
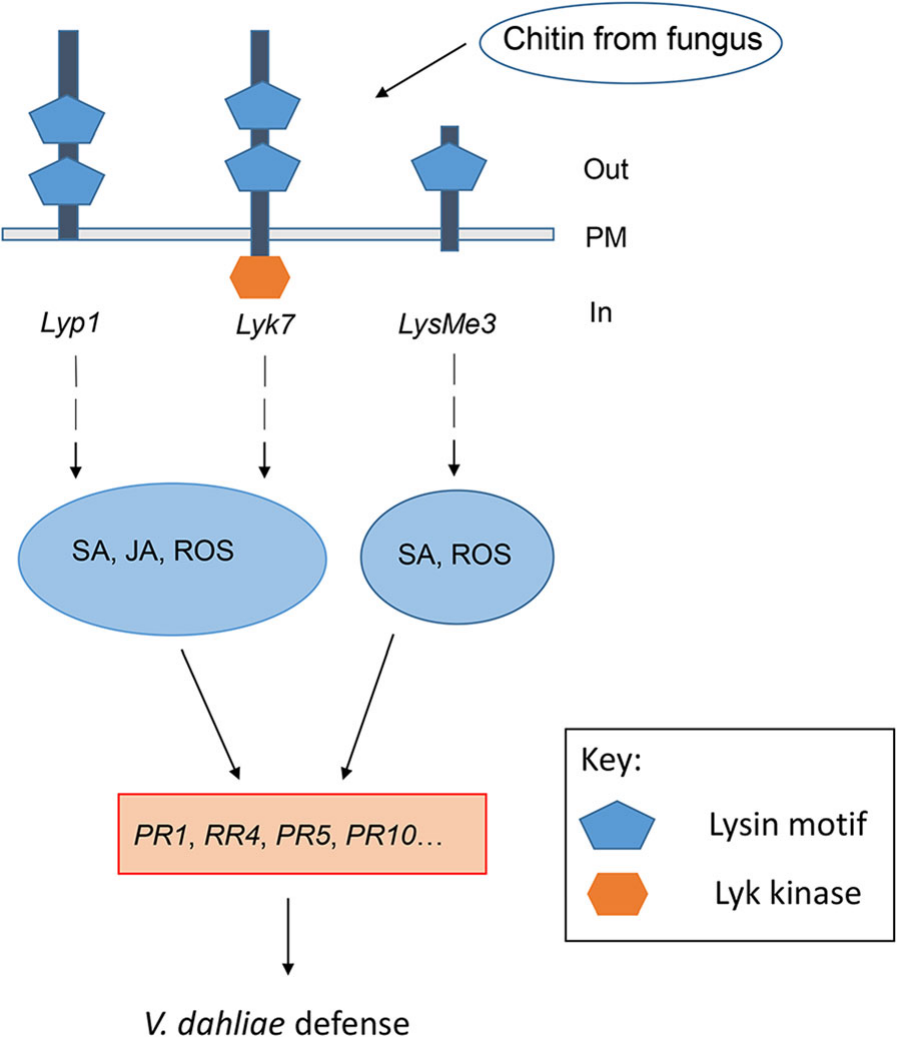
Model for *Lyp1*, *Lyk7*, and *LysMe3* involvement in chitin perception and *Verticillium dahliae* defense. Lyp1, Lyk7, and LysMe3 were found to be membrane-anchored proteins and their expression was induced significantly by chitin signals. *Lyp1*, *Lyk7*, and *LysMe3* not only activated downstream SA, JA, or ROS pathways, but also affected defense gene expression following *V. dahliae* infection. The exogenous application of SA, JA or ROS to cotton plants further promoted the upregulation of these defense genes. PM, plasma membrane

To determine whether *Lyp1*, *Lyk7*, and *LysMe3* silencing affected downstream defense-related genes, we further analyzed the expression of *PR1*, *PR4*, *PR5*, and *PR10* in the VIGS plants. As shown in Fig. 9c, compared with that in TRV: 00 control plants, the expression of the four genes was significantly lower in the *Lyp1*-, *Lyk7*-, and *LysMe3*- silenced cotton plants. We suppose that *Lyp1*, *Lyk7*, and *LysMe3* act as plasma membrane receptors in cotton plants to recognize chitin signals and activate a common downstream pathway, and subsequently induce the expression of related defense genes, therefore enhancing resistance to *V. dahliae* (Fig. 10).

## Discussion

### Genome-wide distribution and characterization of LysM genes in various plant species

LysMs, carbohydrate-binding modules with a length of approximately 40 amino acids, can bind to N-acetylglucosamine (GlcNAc)-containing glycans, such as peptidoglycan, chitin, and chitin-like compounds [44]. These genes therefore usually act as PRRs to detect PAMPs of invading pathogens, and activate defense genes and plant innate immunity. Based on the genomes of several plants, systematic genome-wide investigation of LysM genes has been carried out in these species.

LysM genes are distributed unevenly in the chromosomes in a variety of species [45]. For example, in *Arabidopsis*, 14 LysM genes, 5 *Lyk*s, 3 *Lyp*s, 3 *LysMe*s, and 3 *LysMn*s, are distributed throughout all five of the chromosomes. In *Glycine max*, 47 LysM genes, comprising 21 *Lyk*s, 4 *Lyp*s, 16 *LysMe*s, and 6 *LysMn*s, are also positioned on all chromosomes, expect chromosome 12 (https://phytozome.jgi.doe.gov/pz/portal.html). However, in rice, 20 LysM genes, comprising 6 *Lyk*s, 6 *Lyp*s, 4 *LysMe*s, and 4 *LysMn*s are only positioned on 8 of 12 chromosomes, and in *P. trichocarpa*, 35 LysM genes, comprising 11 *Lyk*s, 7 *Lyp*s, 10 *LysMe*s, and 7 *LysMn*s, are distributed in 12 of 19 chromosomes. The differences in the distribution of LysM genes between species might be related to the individual defense characteristics of each species that developed during the evolutionary process, and interestingly, many duplicated LysM genes also display different expression patterns [10, 45]. Here, we first systematically identified 29, 30, 60, and 56 LysM genes in four sequenced cotton species; the diploid cottons *G. raimondii* and *G. arboreum*, and the tetraploid cottons, *G. hirsutum* acc. TM-1 and *G. barbadense* acc. 3–79, respectively. The 29 LysM genes in *G. raimondii* were anchored to all 12 chromosomes, except Chr. D7, implying that the LysM genes are widely distributed in the *Gossypium* genome. From an evolutionary point of view, we can consider that one member of the LysM gene family in the diploid species *G. raimondii* corresponds to one homologous gene in *G. arboreum* and two homologs from the A and D subgenomes in tetraploid *G. hirsutum* acc. TM-1 and *G. barbadense* acc. 3–79. We found that 14 members of the LysM gene family had such a correspondence in the four sequenced cotton species, indicating that the A- and D-subgenomes evolved independently after polyploid formation (Additional file 1: Table S1). The other inconsistencies may result from chromosome segmental or tandem duplication events during the evolution of different cotton species*,* the sequence quality and type of sequencing methods used in different cotton species, or assembly error in partial chromosomal regions. This requires further investigation.

The secretory pathways, signal peptides and transmembrane domains of proteins are particularly crucial to cellular function during defense against both biotic and abiotic stresses [46]. In this study, characteristics of the LysM genes, including their signal peptides (SPs), subcellular localization, and transmembrane domains were investigated in *G. raimondii* (Additional file 3: Table S2). We found that SPs existed in 15 LysM proteins, suggesting that the proteins are synthesized as pre-proteins, are subsequently cleaved at the signal peptide site to form a mature protein, and function by targeting the general secretary pathway. In addition, 12 LysM proteins, comprising all the GrLyks, GrLyp1, GrLysMe3, GrLysMe9, and GrLysMn7, possessed transmembrane domains; indicating that they have multiple complex functions. In addition to containing transmembrane domains and being involved in secretory pathways, eight LysM proteins, Lyp1–5, Lyk7, Lyk8, and Lyp1, also possessed signal peptides, indicating that they play important roles in the defense against a variety of stresses in cotton.

### LysM genes show multiple expression patterns

Previous reports have shown that the expression of plant LysM genes can be both constitutive and induced [10, 11, 47, 48]. Most LysM kinase genes in *Glycine max* are predominantly expressed in the roots, and orthologous genes have similar tissue expression patterns [10]. In the present study, we systematically analyzed the expression patterns of LysM genes in cotton. Using transcriptome data from *G. hirsutum* acc. TM-1 vegetative and reproductive organs, a total of 50 LysM genes were found to have diverse developmental and spatial regulation patterns (Fig. 2), and most genes showed both diverse and overlapping expression patterns in various tissues and organs, suggesting that these genes have a range of functions but with the identical conserved domains.

There is an increasing volume of data to indicate that LysM-containing proteins can detect several PAMPs, such as the bacterial oligosaccharide, peptidoglycan, and fungal chitin. Upon detection of PAMPs, these proteins then activate a wide range of physiological responses, including the production of SA, JA, ET, and ROS, as well as mitogen activated protein kinase (MAPK) phosphorylation, calcium influx, and the expression of defense-related genes [18, 49]. In *A. thaliana*, *AtCERK1* was shown to be a key chitin receptor, and mediates chitin-induced signaling through homodimerization and phosphorylation [12, 14], and in rice, CEBiP associates with chitin-elicitor receptor kinase 1 (OsCERK1) to mediate MAMP-triggered immunity (MTI) in response to chitin [17, 50]. To better validate the roles of cotton LysM genes in chitin recognition and chitin signal transduction, we analyzed the expression patterns of 20 highly expressed LysM genes in different Hai7124 tissues following treatment with insoluble crab shell chitin and soluble chitin fragment N-acetylchitohexaose. Sixteen genes were significantly upregulated in response to these two PAMPs at different time points, suggesting that LysM proteins play important roles in chitin recognition (Fig. 3). Further, we found that 12 LysM genes, 4 *Lyk*s, 3 *Lyp*s, 1 *LysMe*, and 4 *LysMn*s, had high expression levels in Hai7124 after inoculation with *V. dahliae* strain V991, reaching peak expression levels at different time points (Fig. 4). *Lyp1*, *Lyk7*, *LysMe3*, and *LysMn6* were significantly induced following treatment with both chitin signals and *V. dahliae*. These findings imply that the LysM genes play critical roles in fungal perception and function in cotton defense mechanisms against *V. dahliae*.

The *G. hirsutum* and *G. barbadense* species probably originated from a single hybridization event between A- and D- diploid species, however, the two have very different agronomic and fiber quality characteristics. For example, most of modern *G. barbadense* cultivars are resistance to *V. dahliae*, however, *G. hirsutum* cultivars are not. As described above, we found that *Lyp1*, *Lyk7* and *LysMe3* were significantly upregulated in cotton roots at different time points after inoculation with *V. dahliae*, and had higher expression levels in *G. barbadense* cv. Hai7124 than *G. hirsutum* cv. Junmian 1, indicating that these genes act as positive regulators in plant resistance to *V. dahliae*.

### LysM genes function in the defense against biotic stresses

LysM genes mainly function in recognizing chitin elicitor signals and activating plant immune responses. This has been investigated in various plant species, such as Arabidopsis, wheat, rice, and tomato [15, 17, 47, 51]. In Arabidopsis, CERK1, a LysM receptor kinase, responds to chitin elicitors resulting in MAPK activation and ROS generation [14]. Like CERK1, LYK4 and LYK5 have been localized to the plasma membrane, and are involved in chitin recognition, chitin signal transduction and plant innate immunity [15, 16]. LYM1 and LYM3, two plasma membrane proteins, physically interact with peptidoglycans and mediate immunity to bacterial infection [33]. In rice, a plasma membrane glycoprotein, OsCEBiP, and a receptor kinase, OsCERK1, act as critical components of chitin signaling recognition pathways [17]. Similarly, LYP4 and LYP6 are promiscuous PRRs for PGN and chitin recognition, and are involved in ROS generation, defense gene activation, and callose deposition in rice [18]. In *Medicago*, LYK3 is proposed to function as the entry receptor in rhizobial nodulation factor signaling and specifically controls responses to infection [48]. Taken together, these data show that LysMs are important in the recognition of PGN and chitin signals, and the activation of plant immune responses to biotic stresses.

In this study, cotton *Lyp1*, *Lyk7*, *LysMe3*, and *LysMn6* genes were upregulated at different time points after *V. dahliae* treatment. We observed that *Lyp1*-, *Lyk7*, and *LysMe3*-silenced *G. barbadense* cv. Hai7124 plants had more-severe disease symptoms than the TRV: 00 control and *LysMn6*- silenced plants after *V. dahliae* infection (Figs. 5b and 6b). In addition, statistical analysis suggested that the percentage of diseased leaves in *Lyp1*-, *Lyk7*-, and *LysMe3*-silenced plants was significantly higher than in control plants (Fig. 5c, Additional file 7: Table S3; Fig. 6c, Additional file 8: Table S4). These findings imply that *Lyp1*, *Lyk7*, and *LysMe3*, which have transmembrane domains, function as important chitin receptors, allowing them to recognize *V. dahliae* and induce cotton immunity.

### *Lyp1*, *Lyk7*, and *LysMe3* contribute to *V. dahliae* resistance through the activation of plant innate immune responses

Chitin, a major component of the fungal cell wall, is a typical PAMP and is recognized by PRRs, which activate PTI. PTI is characterized by a wide range of physiological responses, including the production of ROS, calcium influx, and the expression of defense-related genes [4]. In addition, chitin signaling pathways appear to be independent, and different PAMPs might activate a common downstream pathway to induce pathogen resistance [1, 52, 53]. An increasing volume of data further demonstrates that plasma membrane receptors recognize chitin signals and activate downstream SA, JA, ET, and ROS pathways [3, 18, 54].

Chitin binding sites have been identified in the plasma membrane of several plants [5–8]. Here, we analyzed the subcellular location of Lyp1, Lyk7, and LysMe3, and obtained the direct evidence that these are membrane-anchored proteins (Fig. 7). To determine whether *Lyp1*, *Lyk7*, and *LysMe3* affect downstream SA, JA, ET, and ROS production, we examined the expression of *Lyp1*, *Lyk7*, and *LysMe3* after SA, JA, ET, and H_2_O_2_ treatments. Compared to the control, all three genes were significantly induced after SA, JA, and H_2_O_2_ treatment (Fig. 8a). We also examined the expression of several important genes in the SA pathway, *GbEDS1*, *GbPAD4*, *GbEDS5*, and *GbSID2* [35, 36], the JA pathway, *GbAOS*, *GbOPR3*, *GbMYC2*, and *GbJAZ1* [37], and the ROS pathway *GbRBOH*, *GbCAT1*, *GbPOD*, and *GbSOD* [38, 39] in VIGS plants. These genes were significantly down-regulated in the *Lyp1*-, *Lyk7*-, and *LysMe3*-silenced cotton plants compared to the control (Fig. 8b), indicating that the SA, JA, and ROS pathways were perturbed in *Lyp1*-, *Lyk7*-, and *LysMe3*-silenced plants.

PR genes play important roles in signal recognition and plant immunity. For example, in tobacco, *PR1* is involved in the stress response and is associated with resistance to oomycete pathogens [40]. *PR4* encodes chitinase, which is an endogenous plant defense enzyme that generates signaling molecules (elicitors) for the induction of further defenses [41]. In *A. thaliana*, *PR5* is thought to regulate SA biosynthesis and lead to the accumulation of high levels of camalexin in order to protect the plant against pathogen infection [42]. In addition, *PR10* is suggested to increase pepper’s resistance to the oomycete pathogen, *Hyaloperonospora arabidopsidis* [43]. In the present study, we examined the expression of several defense-related marker genes, *PR1*, *PR4*, *PR5*, and *PR10*, in *Lyp1-*, *Lyk7-*, and *LysMe3-*silenced VIGS plants. The four PR genes were significantly upregulated in control plants after *V. dahliae* infection (Fig. 9a); but were all significantly down-regulation in the *Lyp1*-, *Lyk7*-, or *LysMe3*-silenced plants compared to the control, suggesting that defense-related processes were restrained in the VIGS plants, leading to moderate susceptibility to fungal pathogens such as *V. dahliae*. When challenged by pathogens, plant levels of the signaling compounds, SA, JA, ET, and ROS change, PR gene expression is induced and plant resistance to infection is enhanced [55, 56]. In our study, the expression of *PR1*, *PR4*, *PR5*, and *PR10* was significantly increased after SA, JA, or ROS treatment in cotton plants, but was significantly lower in the *Lyp1*-, *Lyk7*-, and *LysMe3*- silenced cotton plants (Fig. 9b, c). Taken together, these findings suggest that cotton LysM-containing proteins are involved in activating downstream defense processes to enhance resistance to *V. dahliae*.

Recent studies have shown that common mechanisms for receptor activation involve receptor homodimerization or oligomerization and subsequent phosphorylation, and that the LysM receptors might function as protein complexes [16]. In Arabidopsis, AtFLS2 was associated with AtBAK1 upon flagellin treatment, which initiated cellular defense signaling [57, 58]. In addition, the AtLYK5-AtCERK1 interaction is chitin dependent, yet AtLYK4 can interact with AtCERK1 independently of the presence of chitin [16]. Here, we confirmed that the three LysM-containing proteins, Lyp1, Lyk7, and LysMe3, act as plasma membrane receptors in cotton and recognize chitin signals, activate downstream SA, JA, and ROS pathways, induce PR gene expression, and enhance resistance to *V. dahliae* (Fig. 10). However, the functional characteristics of *Lyp1*, *Lyk7*, and *LysMe3* in the resistance network remain to be clarified. Future investigations into the effect of the three LysMs on specific defense-related genes and their involvement in the defense network will be meaningful, not only to improve our understanding of the molecular mechanisms of LysM-chitin interactions, but also for developing fungal-resistant cultivars through breeding programs for cotton and other crops.

## Conclusion

Cotton *Verticillium* wilt, a highly destructive vascular disease caused by the soil-borne pathogen *V. dahliae,* leads to devastating reductions in plant mass, lint yield, and fiber quality. Although efforts have been made to produce wilt-resistant cotton cultivars by traditional breeding, to date, *Verticillium* wilt is not effectively controlled in most cotton producing areas. Here, we focused on LysM-containing proteins, which can sense pathogen PAMPs and promote plant defenses. We mined the key LysM genes involved in the cotton defense response to *V. dahliae* infection. Lyp1, Lyk7, and LysMe3 were found to act as plasma membrane receptors that recognize chitin signals, activate downstream defense processes, and enhance resistance to *V. dahliae*. These findings not only clarify the important roles of LysM genes in the defense against *V. dahliae* infection in cotton, but also enrich our knowledge of the networks of plant resistance.

## Methods

### Identification of the LysM genes in four sequenced cotton species

Data were obtained from the genomic databases of four sequenced cotton species, *G. raimondii* (http://www.phytozome.net/), *G. arboreum* (http://cgp.genomics.org.cn), *G. hirsutum* acc. TM-1 (http://mascotton.njau.edu.cn/) and *G. barbadense* acc. 3–79 (http://cotton.cropdb.org/cotton/). The Hidden Markov Model (HMM) profile of the LysM domain (PF01476) was downloaded from the Pfam database (http://pfam.xfam.org/) [59], and this profile then acted as a query to screen all LysM proteins in the sequenced cotton species using HMMER (V3.0) software [60]. The existence of the conserved domains of LysM was further verified in the sequences using SMART [30] and INTERPROSCAN [31].

### Chromosomal localization, phylogenetic tree construction, and characterization analysis

MapInspect software (http://www.softsea.com/review/MapInspect.html) was used to analyze the distribution of LysM genes in *Gossypium*. ClustalX (version 2.0) was used to construct multiple sequence alignments of LysM proteins without gaps and poorly aligned sections. A phylogenetic tree was generated by the MEGA 5.1 software (http://www.megasoftware.net/) using the maximum likelihood method, and the reliability of interior branches was assessed with 1000 bootstrap resampling. The online Gene Structure Display Server (GSDS) program (http://gsds1.cbi.pku.edu.cn/) was used to analyze exon/intron structures through alignment of the genomic DNA sequences with their corresponding coding sequences. In addition, LysM protein motifs were investigated using MEME (http://meme-suite.org/), which set the maximum number of motifs at 8 to further verify the phylogenetic classification of each protein. Signal peptides were predicted using the SignalP 4.1 Server (http://www.cbs.dtu.dk/services/SignalP/). The TargetP 1.1 Server (http://www.cbs.dtu.dk/services/TargetP/) was used to analyze the subcellular localization of LysM proteins and the SMART [30] program was used to analyze the transmembrane domains of LysM genes.

### Plant materials and treatments

The expression of LysM1 genes was analyzed in *G. barbadense* cv. Hai7124, *G. hirsutum* cv. Junmian 1, and *G. hirsutum* cv. Jinmian 19 following different stress treatments. Seedlings were grown in the same controlled environment chamber under the same conditions: a 16 h light/8 h dark cycle at 28 °C for 2 weeks. All necessary permits for collecting Hai7124, Junmian 1 and Jinmian 19 were obtained from Nanjing Agricultural University, Jiangsu Province, China.

Hai7124 and Junmian 1 seedlings, which showed resistance and susceptibility to *V. dahliae*, respectively, were inoculated with the fungal pathogen, *V. dahliae*, using the dip-inoculation method [34]. V991, a highly aggressive and defoliating strain of *V. dahliae*, was obtained from our lab (State Key Laboratory of Crop Genetics & Germplasm Enhancement, Nanjing Agriculture University, Nanjing, China), which was cultured on potato dextrose agar medium (PAD) at 24 °C for 4–5 days, and then transferred to Czapek’s medium for incubation at 25 °C for 5 days [37]. Subsequently, we used deionized water to adjust the concentration to 10^7^ conidia per milliliter for inoculation of the seedlings. Seedling roots were harvested at 0, 24, 48, 96, and 144 h after V991 treatment.

Hai7124 seedlings were also treated with 200 mg/mL of insoluble crab shell chitin and soluble chitin fragment N-acetylchitohexaose (Seebio, Shanghai, China). The insoluble crab shell chitin and soluble chitin fragment N-acetylchitohexaose were dissolved in 1% acetic acid and deionized water, respectively. Next, the leaves of Hai7124 seedlings were sprayed with chitin and N-acetylchitohexaose, and leaves were harvested at 0, 0.5, 1, 2, 4, and 6 h following treatment.

*G. hirsutum* cv. Jinmian 19 seedlings, which have a high tolerance to abiotic stresses, were treated with four solutions of defense-related signaling molecules, containing 100 μM JA, 1 mM ET, 100 mM SA and 10 mM H_2_O_2_, respectively. ddH_2_O was used as a solvent control, and leaves were collected at 0, 1, 4, 12, and 24 h after treatment.

For each treatment, three biological repeats were harvested, then quick-frozen in liquid nitrogen and stored at −80 °C before RNA extraction.

### RNA isolation and expression pattern analysis

Total RNA was isolated from cotton seedling leaves and roots using the CTAB-acidic phenolic method [61], and the RNA samples (2 μg per reaction) were reversely transcribed into cDNA using the HiScript Q RT SuperMix for qPCR (+gDNA wiper) (Vazyme).

The gene-specific primers for qRT-PCR analysis were designed using Beacon Designer 7.0. Cotton histone3 (AF024716) was used as the reference gene. All primer information is available in Additional file 9: Table S5. Real-time PCR amplification reactions were performed on an ABI 7500 Real Time PCR System (Applied Biosystems, USA) using AceQ SYBR Green Master (Low Rox Premixed) (Vazyme) with three technical replicates for each biological sample. Expression data from three biologically independent experiments were analyzed and presented as means ± S.D.

*G. hirsutum* acc. TM-1 high-throughput RNA-sequencing data from Zhang et al. [28] was used to systematically analyze the expression patterns of LysM genes in different tissues, including vegetative tissues (root, stem, and leaf), floral tissues (petal and stamen), ovule tissues (−3, 0, and 3 DPA), and fiber tissues (5, 10, 20, and 25 DPA). Subsequently, the Log_2_ (FPKM) formula was used to calculate and analyze the expression levels of the LysM genes, where FPKM refers to fragments per kilobase of exon model per million mapped reads as identified using Cufflinks software (http://cufflinks.cbcb.umd.edu/). A heat map was generated with Multi Experiment Viewer v. 4.9 (http://en.bio-soft.net/chip/MeV.html).

For statistical analysis, all generated data in this study were repeated at least three times on three biological replicates. The data from three biologically independent experiments were analyzed and presented as means ± S.D. In addition, statistical significance was determined by Student’s *t*-tests.

### Cloning of the LysM genes in *G. barbadense* cv. Hai 7124

Based on the known sequences of four cotton species, gene-specific primers were designed using Primer 5.0 software to amplify the homologous genes of LysM with complete open reading frames (ORFs) in *G. barbadense* cv. Hai7124, a cultivar resistant to *V. dahliae* (Additional file 9: Table S5). High-fidelity ExTaq DNA Polymerase (TaKaRa Biotechnology [Dalian] Co. Ltd., China) was used in standard PCR reactions. All PCR products were cloned into pMD19-T cloning Vectors (TaKaRa, Dalian, China) and transformed into bacterial strains *E.coli* DH5α that was obtained from our lab (State Key Laboratory of Crop Genetics & Germplasm Enhancement, Nanjing Agriculture University, Nanjing, China). At least six clones per gene were randomly selected and sequenced.

### Vector construction and functional characterization of candidate genes via virus-induced gene silencing (VIGS)

The pTRV1 and pTRV vectors used for VIGS analysis were generously provided by Dr. Libo Shan of Texas A & M University (College Station, TX, USA). *GhCLA1* (Cloroplastos alterados 1), which encodes 1-deoxy-D-xylulose-5-phosphate synthase, was used to construct a pTRV: *GhCLA1* vector that acted as a control to verify the efficiency of the VIGS procedure [62]. TRV vectors were constructed to silence target genes: TRV: *Lyk7* contained a 400 base pair (bp) gene-specific fragment of *Lyk7* cDNA, TRV: *Lyp1* included a 447 bp fragment of *Lyp1*, TRV: *LysMe3* contained the full-length 312 bp sequence of *LysMe3*, and TRV: *LysMn6* included a 494 bp gene-specific fragment of *LysMn6*. The primers used for constructing the VIGS vectors are shown in Additional file 9: Table S5.

These vectors were transformed into *Agrobacterium tumefaciens* strain GV3101 that was obtained from our lab (State Key Laboratory of Crop Genetics & Germplasm Enhancement, Nanjing Agriculture University, Nanjing, China), and the *Agrobacteria* containing TRV1 were subsequently mixed with TRV: 00, TRV: *GhCLA1*, TRV: *Lyp1*, TRV: *Lyk7*, TRV: *LysMe3*, and TRV: *LysMn6* at a 1:1 ratio, before being infiltrated into the fully expanded cotyledons of eight-day-old Hai7124 cotton seedlings [63]. All plants were grown in the same growth chamber at 23/21 °C (day/night), with a 16 h light/8 h dark cycle and changes in plant phenotypes were observed. Two weeks after *Agrobacterium* infiltration, the TRV: *GhCLA1* plants showed highly uniform bleaching in newly emerged leaves. We harvested the leaves from at least three seedlings per treatment and isolated their RNA. All VIGS cotton seedlings, as well as the susceptible control Junmian 1, and the resistant control Hai7124 without *Agrobacterium* infiltration, were removed from the soil and dip-inoculated with V991 conidia suspension (1 × 10^7^conidia/mL) as described previously [34]. Subsequently, all plants were grown in the same growth chamber at 25/23 °C (day/night), with a 16 h light/8 h dark cycle for 7 weeks and the ratio of diseased to healthy leaves was analyzed. Each treatment was applied to more than 20 plants and the VIGS experiments were repeated at least three times to increase the reliability of the results. Statistical analyses were used to compare the percentage of diseased leaves in experimental plants with that in TRV: 00 controls.

### Subcellular localization of *Lyp1*, *Lyk7*, and *LysMe3* in onion epidermal cells

The ORFs of the *Lyp1*, *Lyk7*, and *LysMe3* genes were fused to green fluorescent protein (GFP) under the control of the 35S promoter in the pBin-GFP4 expression vector [64]. Plasmids harboring GFP alone were used as controls. The onions were bought in the vegetable supermarket and the onion epidermal cells were cultured in MS culture medium at 24 °C for 3 h in the lab. Plasmid DNA at a concentration of 1 μg/ μL or greater was used for biolistic bombardment. Helios Gene Gun (Bio-Rad) was used to bombard plasmid DNA into onion cells. After transformation, the onion cells were incubated at 25 ± 1 °C for 16 h in dark conditions. Fluorescent signals were visualized with a confocal microscope (Zeiss, LSM710; Germany), and 20% sucrose solution was used for the plasmolysis of the onion cells.

## Additional files

Additional file 1: Table S1. Identification of the LysM genes in *G. raimondii* and their phylogenetic relationships with those in *G. arboreum*, *G. hirsutum* and *G. barbadense*. (XLS 39 kb)

Additional file 2: Figure S1. Phylogenetic classification and structural analysis of LysM members in *G. raimondii.* The 8 motif components and gene structures (exon-intron organizations) of the four LysMs groups: (A) *Lyk*s, (B) *Lyp*s, (C) *LysMe*s, and (D) *LysMn*s. The gene structures were obtained in accordance with the phylogenetic classifications. (TIFF 871 kb)

Additional file 3: Table S2. Prediction of the signal peptides, subcellular localization, and transmembrane domains of LysM genes in *G. raimondii*. (XLS 32 kb)

Additional file 4: Figure S2. Silencing of the endogenous Cloroplastos alterados gene (*GbCLA1*) in cotton through tobacco rattle virus (TRV)-mediated virus-induced gene silencing (VIGS). Eight-day-old cotton seedlings (Hai7124) with two fully expanded cotyledons were infiltrated with TRV: GbCLA1, and the leaf bleaching phenotype was observed 2 weeks later. (TIFF 860 kb)

Additional file 5: Figure S3. Disease symptoms in *G. barbadense* cv. Hai 7124 and *G. hirsutum* cv. Junmian 1. *G. barbadense* cv. Hai 7124 (resistant) and *G. hirsutum* cv. Junmian 1 (susceptible) seedlings were grown in the same environment and dip-infected with the liquid containing *V. dahliae* strain V991 spores. Control plants were treated with sterile distilled water as a mock treatment. Disease symptoms 20 d and 25 d after infection are shown, and almost 25 days later, all Junmian 1 plants were defoliated. (TIFF 1153 kb)

Additional file 6: Figure S4. The expression of LysM genes from the same group in control and VIGS plants. qRT-PCR analysis was used to confirm the expression of LysM genes from the same phylogenetic group as the silenced gene in control and VIGS plants. Only 4 *Lyk*s, 3 *Lyp*s, 3 *LysMe*s, and 3 *LysMn*s were detected in leaf tissue of the corresponding VIGS plants, with no significant differences in expression levels between the control and VIGS plants. (TIFF 159 kb)

Additional file 7: Table S3. Percentage of wilted leaves in TRV: *GbLyp1 and* TRV: *GbLyk7* treated plants after *V. dahliae* inoculation. (DOC 32 kb)

Additional file 8: Table S4. Percentage of wilted leaves in TRV: *GbLysMe3 and* TRV: *GbLysMn6* treated plants after *V. dahliae* inoculation. (DOC 33 kb)

Additional file 9: Table S5. Information on the PCR primers used in this study. (XLS 36 kb)

## Abbreviations

AA: Amino acids
bp: Base pair
DPA: Days post anthesis
ET: Ethylene
H_2_O_2_: Hydrogen peroxide
JA: Jasmonic acid
LysM: Lysin motif
PAMP: Pathogen associated molecular pattern
PGN: Peptidoglycan
PRR: Pattern recognition receptor
PTI: PAMP-triggered immunity
qRT-PCR: Quantitative real time-PCR
ROS: Reactive oxygen species
SA: Salicylic acid
VIGS: Virus-induced gene silencing
