# Supplementary figures and images for: The lysin motif-containing proteins, Lyp1, Lyk7 and LysMe3, play important roles in chitin perception and defense against Verticillium dahliae in cotton
Source: BMC Plant Biol. 2017 Sep 4;17:148. doi: 10.1186/s12870-017-1096-1 (PMC5583995; doi:10.1186/s12870-017-1096-1)

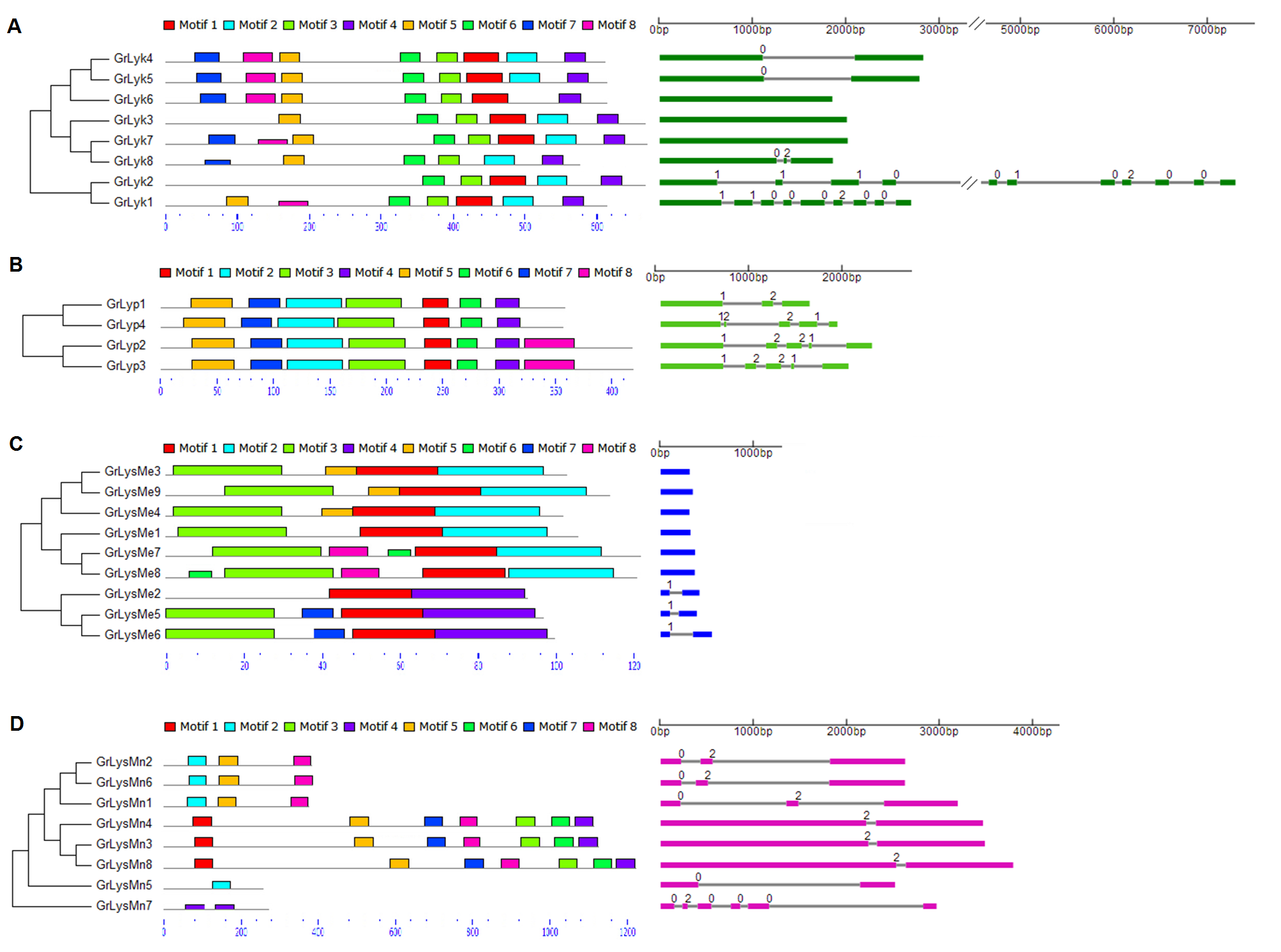

Supplement: Supplementary file 2 — Phylogenetic classification and structural analysis of LysM members in G. raimondii. The 8 motif components and gene structures (exon-intron organizations) of the four LysMs groups: (A) Lyks, (B) Lyps, (C) LysMes, and (D) LysMns. The gene structures were obtained in accordance with the phylogenetic classifications. (TIFF 871 kb) [file 12870_2017_1096_MOESM2_ESM.tif]

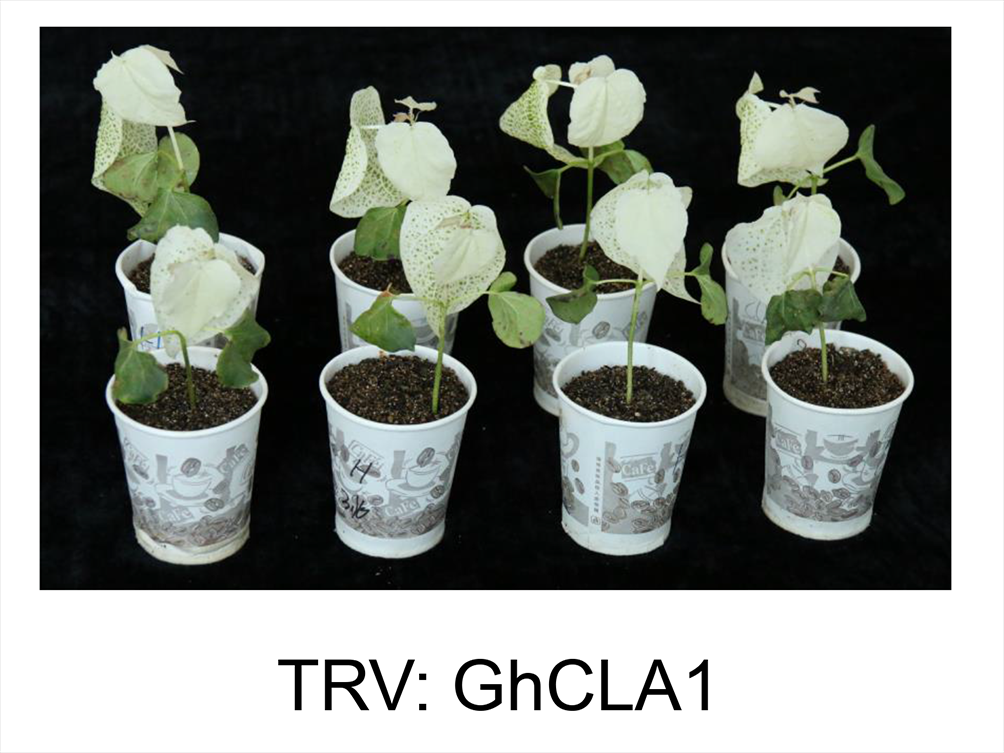

Supplement: Supplementary file 4 — Silencing of the endogenous Cloroplastos alterados gene (GbCLA1) in cotton through tobacco rattle virus (TRV)-mediated virus-induced gene silencing (VIGS). Eight-day-old cotton seedlings (Hai7124) with two fully expanded cotyledons were infiltrated with TRV: GbCLA1, and the leaf bleaching phenotype was observed 2 weeks later. (TIFF 860 kb) [file 12870_2017_1096_MOESM4_ESM.tif]

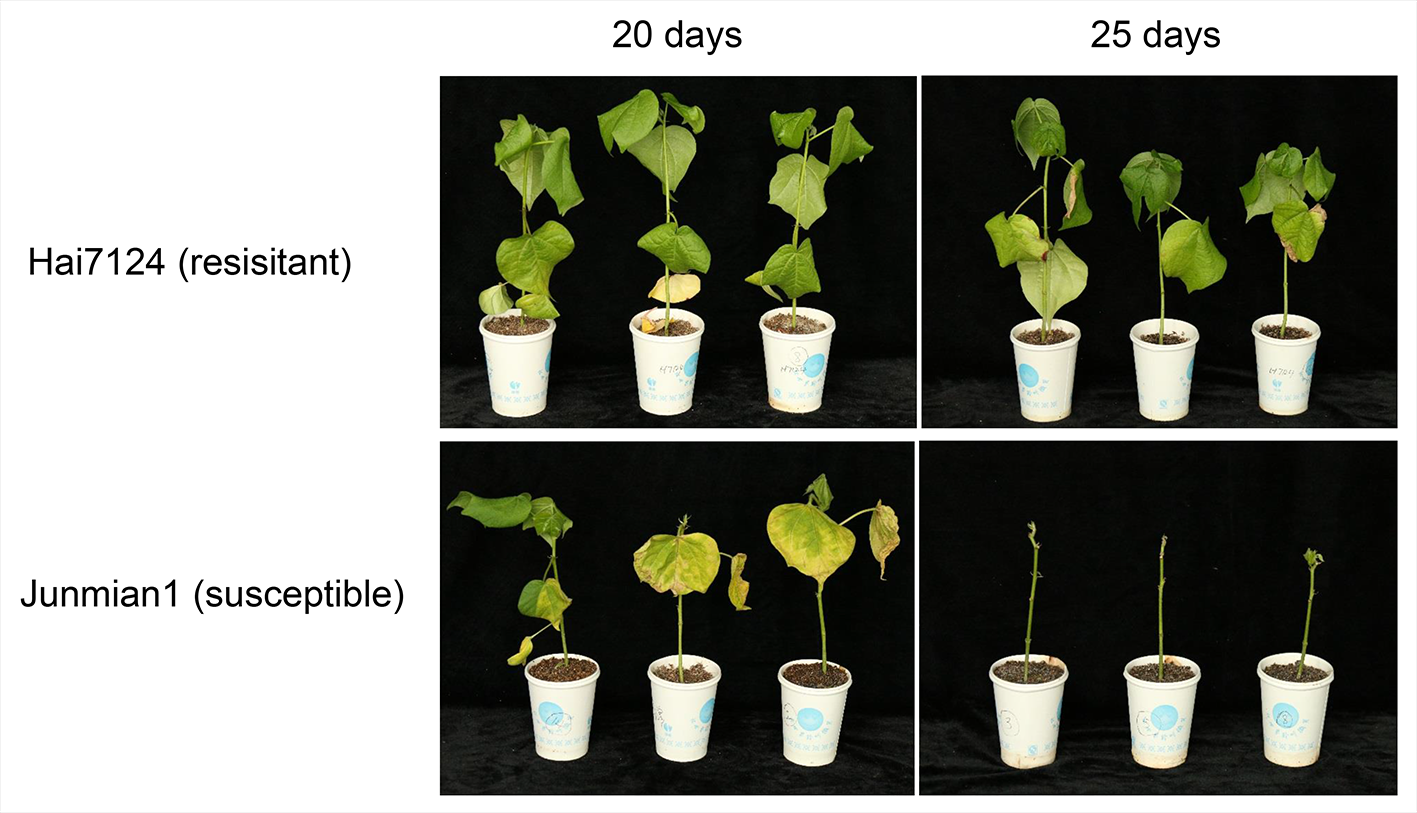

Supplement: Supplementary file 5 — Disease symptoms in G. barbadense cv. Hai 7124 and G. hirsutum cv. Junmian 1. G. barbadense cv. Hai 7124 (resistant) and G. hirsutum cv. Junmian 1 (susceptible) seedlings were grown in the same environment and dip-infected with the liquid containing V. dahliae strain V991 spores. Control plants were treated with sterile distilled water as a mock treatment. Disease symptoms 20 d and 25 d after infection are shown, and almost 25 days later, all Junmian 1 plants were defoliated. (TIFF 1153 kb) [file 12870_2017_1096_MOESM5_ESM.tif]

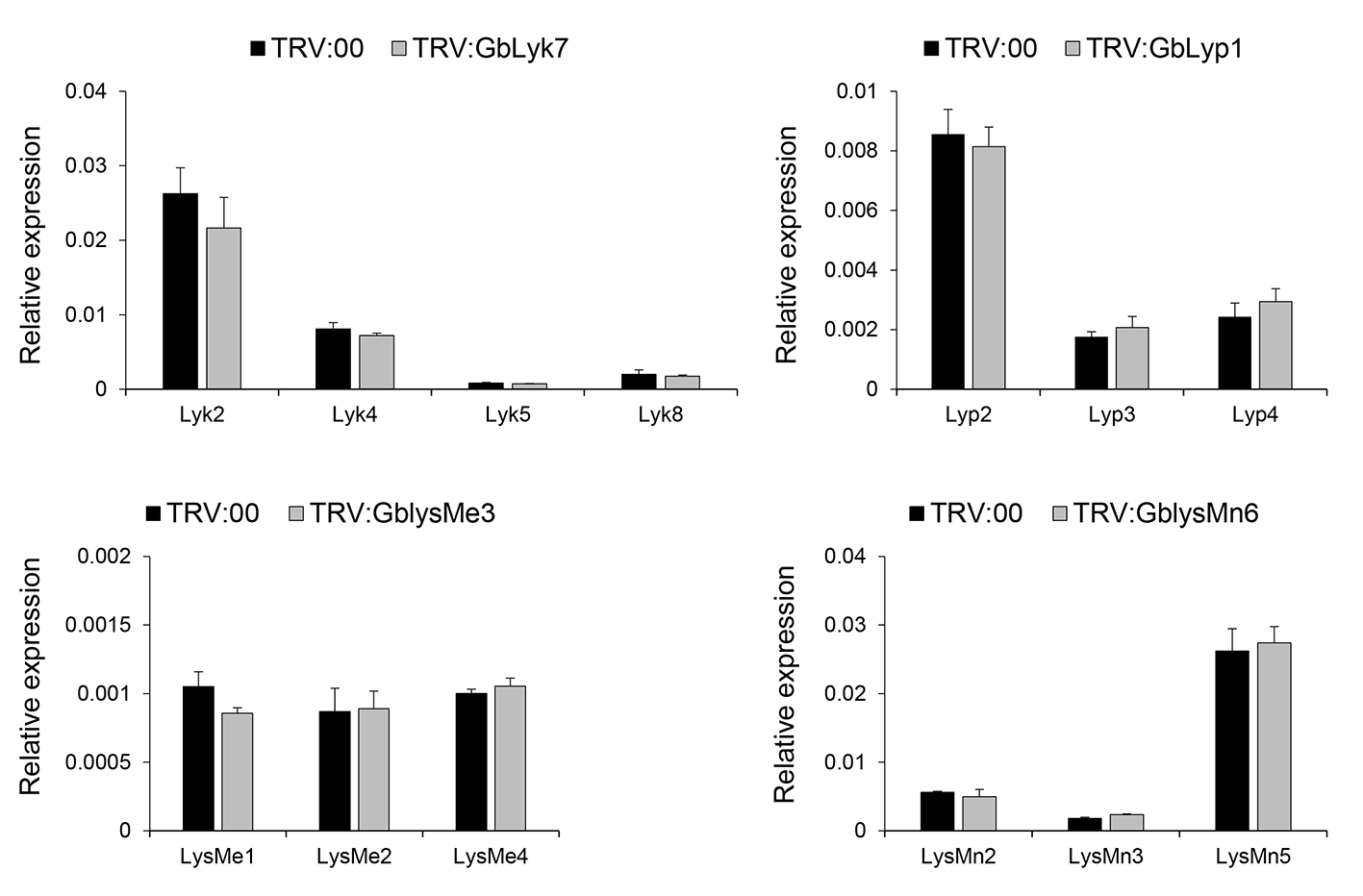

Supplement: Supplementary file 6 — The expression of LysM genes from the same group in control and VIGS plants. qRT-PCR analysis was used to confirm the expression of LysM genes from the same phylogenetic group as the silenced gene in control and VIGS plants. Only 4 Lyks, 3 Lyps, 3 LysMes, and 3 LysMns were detected in leaf tissue of the corresponding VIGS plants, with no significant differences in expression levels between the control and VIGS plants. (TIFF 159 kb) [file 12870_2017_1096_MOESM6_ESM.tif]
