# Supplementary material for: The lysin motif-containing proteins, Lyp1, Lyk7 and LysMe3, play important roles in chitin perception and defense against Verticillium dahliae in cotton
Source: BMC Plant Biol. 2017 Sep 4;17:148. doi: 10.1186/s12870-017-1096-1 (PMC5583995; doi:10.1186/s12870-017-1096-1)
Supplement: Supplementary file 7 — Percentage of wilted leaves in TRV: GbLyp1 and TRV: GbLyk7 treated plants after V. dahliae inoculation. (DOC 32 kb) [file 12870_2017_1096_MOESM7_ESM.doc]

| **Treatment** | **Days after *V. dahliae* inoculation** | | | | | | |
| --- | --- | --- | --- | --- | --- | --- | --- |
|  | 11 | 15 | 20 | 25 | | 30 | 35 |
| **Junmian 1** | 21.4±3.8 A | 47.4±4.7 A | 69.7±3.6 A | | 82.4±5.3 A | 94.8±4.4 A | 100±0.0 A |
| **Hai7124** | 0.0±0.0 B | 11.6±4.0 B | 26.2±3.0 B | | 45.8±4.1 B | 51.7±4.0 B | 55.4±5.0 B |
| **TRV:00** | 0.0±0.0 B | 15.5±4.0 B | 30.8±4.0 BC | | 42.8±7.0 B | 55.1±6.0 BC | 56.5±3.0 B |
| **TRV:*GbLyp1*** | 17.1±3.1 A | 33.4±3.1 A | 55.5±4.1 A | | 63.3±3.0 A | 73.2±4.1 C | 87.1±3.1 A |
| **TRV:*GbLyk7*** | 16.4±5.1 A | 35.8±6.1 A | 51.7±5.0 C | | 68.1±4.1 A | 75.0±4.1 A | 86.2±3.1 A |

**Table S3 Percentage of wilted leaves of the TRV: *GbLyp1 and TRV: GbLyk7* after *V. dahliae* inoculation**

The *GbLyp1* and *GbLyk7* were further analyzed by the VIGS method. The experiments were repeated at least three times and each treatment was applied to more than 20 plants to increase the reliability of the results. An average value of “percentage of wilted leaves” was calculated, and the standard deviation reflected the differences among the three independent biological experiments. The statistical significance was determined by Student’s t-tests (P<0.01).
