# Supplementary material for: The lysin motif-containing proteins, Lyp1, Lyk7 and LysMe3, play important roles in chitin perception and defense against Verticillium dahliae in cotton
Source: BMC Plant Biol. 2017 Sep 4;17:148. doi: 10.1186/s12870-017-1096-1 (PMC5583995; doi:10.1186/s12870-017-1096-1)
Supplement: Supplementary file 8 — Percentage of wilted leaves in TRV: GbLysMe3 and TRV: GbLysMn6 treated plants after V. dahliae inoculation. (DOC 33 kb) [file 12870_2017_1096_MOESM8_ESM.doc]

**Table S4. Percentage of wilted leaves of the TRV: *GbLysMe3 and* TRV: *GbLysMn6* after *V. dahliae* inoculation.**

| **Treatment** | **Days after *V. dahliae* inoculation** | | | | | |
| --- | --- | --- | --- | --- | --- | --- |
|  | 11 | 15 | 20 | 25 | 30 | 35 |
| **Junmian 1** | 25.0±3.0 A | 40.9±8.8 A | 71.4±5.6 A | 89.2±4.8 A | 93.0±1.1 A | 100±0.0 A |
| **Hai7124** | 0.0±0.0 B | 3.2±3.5 B | 25.7±6.3 B | 40.9±9.5 BC | 43.5±1.4 C | 47.4±2.2 D |
| **TRV:00** | 0.0±0.0 B | 3.5±2.9 B | 20.2±2.0 B | 41.0±9.4 B | 44.3±3.0 C | 47.2±2.8 D |
| **TRV:*GbLysMe3*** | 0.0±0.0 B | 10.0±1.0 B | 38.4±5.5 B | 57.2±3.6 C | 74.8±5.3 B | 82.5±3.1 B |
| **TRV:*GbLysMn6*** | 0.0±0.0 B | 0.0±0.0 B | 22.2±8.6 B | 41.6±9.8 B | 52.9±8.0 C | 56.8±7.5 C |

The VIGS technology was used for functional analysis of the *GbLysMe3* and *GbLysMn6.* The experiments were repeated three times and each treatment was applied to more than 20 plants to better validate the results. The average value of “percentage of wilted leaves” was calculated, and the standard deviation reflected the differences among the three independent biological experiments. The statistical significance was determined by Student’s t-tests (P<0.01).
